# DNA Vaccination Against Macrophage Migration Inhibitory Factor Protecting Mice From Experimental Pancreatitis

**DOI:** 10.7759/cureus.102441

**Published:** 2026-01-27

**Authors:** Tatsuya Ohkawara, Naoto Okubo, Shunsuke Ohnishi

**Affiliations:** 1 Laboratory of Molecular and Cellular Medicine, Hokkaido University Faculty of Pharmaceutical Sciences, Sapporo, JPN

**Keywords:** cerulein, experimental pancreatitis, heat shock protein, machophage migration inhibitory factor, vaccination

## Abstract

Objective

Macrophage migration inhibitory factor (MIF) has a pivotal role in the development of gastroenterological diseases, including pancreatitis. In this study, we aimed to explore the effect of deoxyribonucleic acid (DNA) vaccination producing an auto-MIF antibody on experimental pancreatitis and to provide additional evidence that MIF affects the development of pancreatitis.

Methods

Mice were treated with an MIF-DNA vaccine by introducing oligonucleotides encoding a helper T epitope into the cDNA sequence of murine MIF by in vivo electroporation. Thereafter, experimental pancreatitis was induced by seven repeated intraperitoneal injections of cerulein (50 μg/kg). Histological findings were evaluated in the pancreas. The levels of MIF, monocyte chemoattractant protein-1 (MCP-1), IL-1β, and heat shock protein 70 (HSP70) were analyzed with enzyme-linked immunosorbent assay (ELISA).

Results

The titer of MIF antibody was increased in the serum of mice 8 weeks after the treatment with MIF-DNA vaccine. In cerulein-induced pancreatitis, the histological findings in the pancreas were ameliorated in MIF-DNA vaccinated mice. The serum levels of MIF were lower in MIF-DNA vaccinated mice than those in mock-treated mice with pancreatitis. The increases in the serum and pancreatic levels of IL-1β and the serum level of MCP-1 were suppressed in MIF-DNA-vaccinated mice given cerulein. Furthermore, the pancreatic HSP70 level was upregulated in MIF-DNA-vaccinated mice given cerulein.

Conclusion

MIF-DNA vaccination protected mice from cerulein-induced pancreatitis via anti-inflammatory and cytoprotective effects. MIF-DNA vaccination may be an additional option for the treatment of pancreatitis.

## Introduction

Pancreatitis is clinically characterized by tissue injury and inflammatory infiltration in the pancreas, and the number of patients with pancreatitis has been increasing [1]. Severe pancreatitis often leads to multiple organ failures and death due to its complications [1]. Although various treatments for pancreatitis have been clinically used, some cases are resistant to current therapies. The mortality rate of patients with severe pancreatitis has been relatively high among benign gastrointestinal diseases [1]. Thus, another therapeutic option is expected.

The mechanism underlying the development of pancreatitis has been well investigated. Proinflammatory cytokines such as TNF-α play an important role in the development of pancreatitis [2,3]. In addition, inhibition of cytokines reduces the severity of experimental pancreatitis [4]. Macrophage migration inhibitory factor (MIF) acts as an enhancer of the immune response and inflammatory processes, resulting in the development of inflammatory diseases [5,6]. MIF is ubiquitously expressed in immunocytes and non-immunocytes, such as epithelial cells, under normal conditions, and its expression is upregulated in response to various stimuli [6]. Furthermore, several experimental disease models, including arthritis, hepatitis, and inflammatory bowel disease, were ameliorated by MIF inhibition [7-9]. In pancreatitis, MIF is upregulated in the blood and tissues in patients [10-12]. Moreover, in vivo studies have shown that the deletion of MIF reduces inflammation and tissue injury in experimental pancreatitis in mice [13].

To neutralize the upregulation of cytokines, the administration of neutralizing antibodies against these cytokines is effective and clinically used in various inflammatory diseases with resistance to pharmacotherapeutic agents. However, there are a few limitations in the treatment with neutralizing antibodies. Repeated treatment with a neutralizing antibody leads to the reduction of the effect of the neutralizing anti-antibody [14]. In addition, the economic cost of treatment with neutralizing antibodies is high. To resolve these problems, Onodera et al. developed a DNA vaccine that encoded the cDNA sequence of the cytokine murine MIF (mMIF) [15]. To enhance the adjuvant effect in the DNA vaccine, the oligonucleotides encoding helper T (TH) epitope were added to the DNA sequence. Previous studies, including our study, demonstrated that MIF-DNA vaccination suppressed the severity of arthritis, dermatitis, and colitis [15-17]. Thus, we hypothesized that MIF-DNA vaccination is effective for the treatment of pancreatitis. In this study, to clarify the potential of the effect of MIF-DNA vaccine in pancreatitis, we evaluated its effect on cerulein-induced pancreatitis in mice.

## Materials and methods

Animals

Five-week-old male BALB/c mice were purchased from Japan CLEA (Shizuoka, Japan) and bred under specific pathogen-free conditions. Mice had free access to the regular chow and water ad libitum, and were kept in the room at a comfortable room temperature (22-24°C) on a 12-hour light and dark cycle. All animal procedures in this study were approved by the Hokkaido University Institutional Animal Care and Use Ethics Committee (No.13-0181) and conducted according to an approved protocol. Mice at six weeks of age were used in the experiments of vaccination.

Research methods

Construction of MIF-DNA Vaccine

MIF-DNA vaccine was constructed as previously described [15]. In brief, a coding region for the second loop of murine MIF, which is coded in the amino acids 32-37. (GKPAQY), was substituted with a cDNA coding for tetanus toxin (TTX) P30 TH epitope (FNNFTVSFWLRVPKVSASHL). This mutant MIF cDNA, in which the second loop region was replaced by TTX, was inserted into the pCAGGS mammalian expression vector and cloned. The plasmid DNA was purified by the standard method using alkaline lysis followed by two rounds of CsCl density gradient ultracentrifugation. The DNA purified from only the pCAGGS vector was used as a control.

Inoculation of MIF DNA Vaccine

Immunization with MIF-DNA vaccine in mice was performed as described previously [15]. Briefly, mice at six weeks of age were temporally anesthetized with diethyl ether and shaved around their bilateral hind legs. A pair of electrode needles (5 mm gap and 0.5 mm diameter, Nepa Gene, Chiba, Japan) was gently inserted into an anterior tibial muscle, and MIF-DNA vaccine (25 μg/25 μL of 0.9% saline) was injected into the portion between the needles. Electric pulses (50 V, 50 msec, three times) were given to mice with an electric pulse generation system (T820 and Optimizer 500, BTX, San Diego, CA), followed by the other pulses with inverted polarity. In addition, the same injection and electroporation were applied to the other tibial muscle. A total of 50 μg of the naked plasmid was injected per mouse in two tibias. DNA vaccination was performed only once. To determine the titer of anti-MIF antibody, eight weeks after vaccination, a small amount of blood sample was obtained from the tail vein. The serum was isolated by centrifugation (3000 g, five minutes) of the blood sample, and the serum anti-MIF antibody formation was measured with our constructed enzyme-linked immunosorbent assay (ELISA) kit (R&D Systems Inc., Minneapolis, MN) as described previously [15].

Induction and Assessment of Experimental Pancreatitis

Eight weeks after MIF-DNA vaccination, experimental pancreatitis was induced by cerulein administration. Mice were divided into four groups as follows: (1) mice treated with only the vector, (2) mice treated with only the MIF-DNA vaccine, (3) mice treated with the vector and cerulein, and (4) mice treated with the MIF-DNA vaccine and cerulein. With reference to the previous reports, experimental pancreatitis was induced by seven times hourly intraperitoneal injection with 50 μg/kg of cerulein in phosphate buffer saline (PBS) [18]. Blood and pancreas were obtained from mice at 12 hours after the initial injection of cerulein. Prior to removing tissues and blood, mice were euthanized by intraperitoneal injection with a sufficient dose of thiopental (Kyoritsu Seiyaku Co., Tokyo, Japan). Then, a blood sample was obtained from the insertion of a catheter into the jugular vein. The samples of serum were isolated by centrifugation (3000 g, five minutes) from blood samples. On the other hand, the pancreas tissue was removed and divided for morphological evaluation and molecular analyses. Parts of the samples for morphological evaluation were stored in 10% neutral buffered formalin solution, and the other parts of the samples were immediately frozen using liquid nitrogen and stored at -20°C until use.

Histological Evaluation of Pancreatitis and Lung Injury

The tissues fixed with 10% neutral buffered formalin were embedded in paraffin and sliced thinly (3 mm). After deparaffinizing the thin sections and putting them on glass slides, the slide samples were stained with H&E. Histological evaluation was performed at a light microscope. Histological severity of pancreatic damage was scored in the field of interstitial edema, inflammatory infiltration, hemorrhage, and acinar cell necrosis using the scoring system as described previously [19].

Measurement of Serum Amylase and Lipase Activities

Amylase and lipase activities in serum were determined with the amylase activity kits (BioVision Inc., Milpitas, CA) and lipase activity kits II (Sigma-Aldrich, St. Luis, MO) according to protocol.

Measurement of Macrophage Migration Inhibitory Factor

The MIF levels of serum and pancreas tissue were measured with the MIF-ELISA kit (Abcam, Cambridge, UK) according to protocol. The concentration of protein weight in supernatant isolated from tissue homogenate was measured with the BCA protein assay kit according to the manufacturer’s instructions (Thermo Scientific Inc., Waltham, MA).

Measurement of IL-1β and Monocyte Chemoattractant Protein (MCP-1)

The levels of IL-1β and MCP-1 in serum were measured with an ELISA kit according to the manufacturer’s instructions. The levels of IL-1β in pancreas tissue were measured with ELISA kits (IL-1β, R&D Systems Inc., Minneapolis, MN) according to the manufacturer’s protocol.

ELISA for Heat Shock Protein 70 in the Pancreas

The pancreas tissue in lysis buffer was homogenized, and the supernatant was isolated from the homogenate by centrifugation for five minutes at 10,000g. The protein levels of HSP70 in the supernatant were determined with ELISA kits for HSP70 (Enzo Biochem Inc., New York, NY) according to the manufacturer’s protocol.

Statistics

All data are presented as mean ± standard error (SE). The results from all experiments were statistically analyzed using analysis of variance (ANOVA) and Tukey’s test (GraphPad Prism 9, GraphPad Software Inc., San Diego, CA). p < 0.05 was considered statistically significant.

## Results

Effect of MIF-DNA vaccination on the titer of anti-MIF antibody in serum

**Figure 1:**
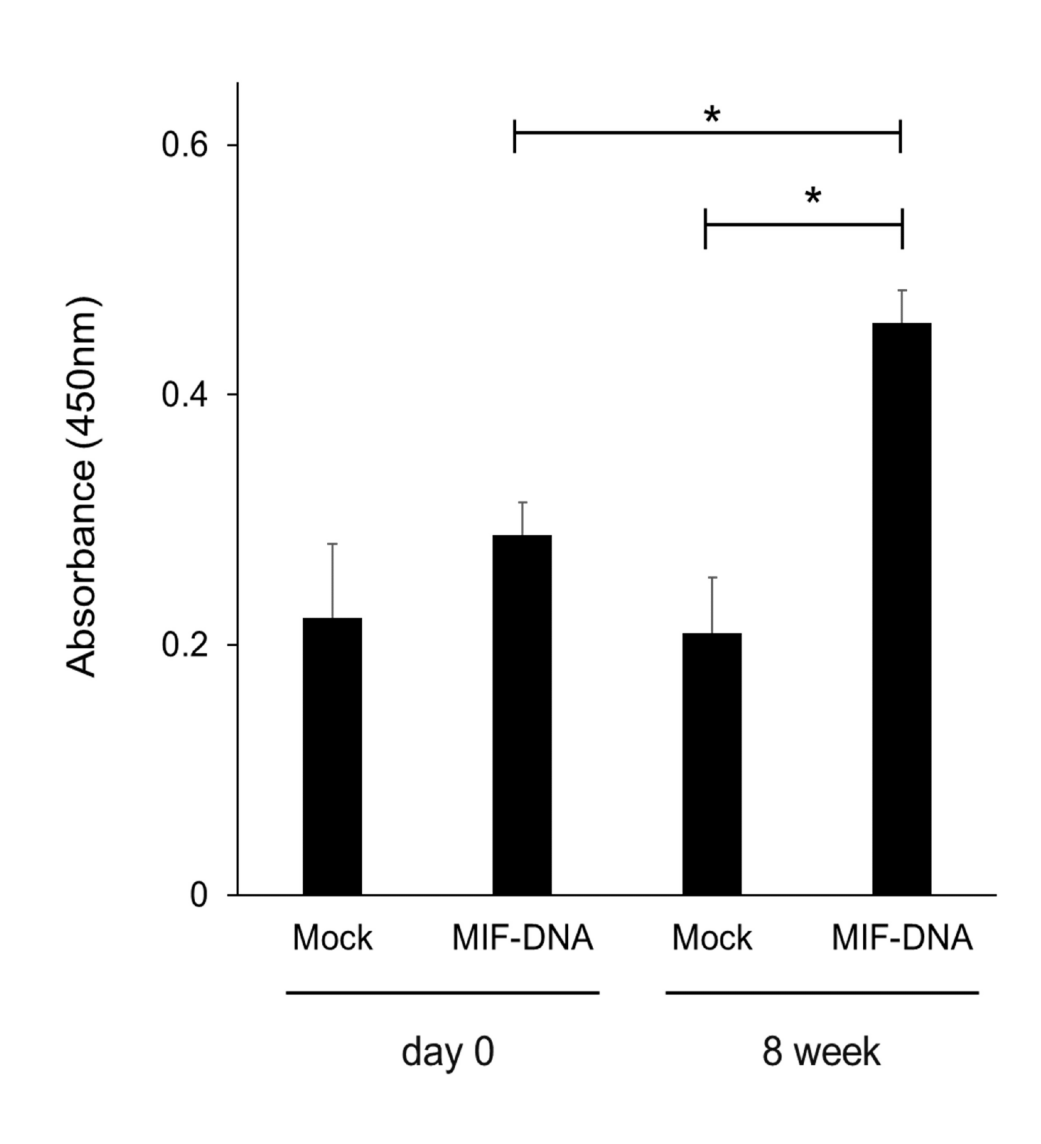
Anti-MIF antibody induction by DNA vaccination and cross-reactivity with native murine MIF. Each group: n = 5. Mock: mock-vaccinated mice, MIF-DNA vaccine: MIF-DNA vaccinated mice. Results were shown as means ± SE. *p < 0.05. MIF, migration inhibitory factor

As shown in Figure 1, the absorbance at the wavelength of 450 nm was raised in the serum of MIF-DNA vaccinated mice compared to mock-vaccinated mice eight weeks after vaccination. On the other hand, just before MIF-DNA vaccine treatment, the levels of MIF antibody titers were similar in both groups. With reference to the previous reports [15-17], our results suggest that the titer of immunoglobulin reacted to MIF in serum was significantly increased in MIF-DNA vaccinated mice (p < 0.05) compared with mock-vaccinated mice.

Evaluation of MIF-DNA vaccination on the severity of cerulein-induced pancreatitis

**Figure 2:**
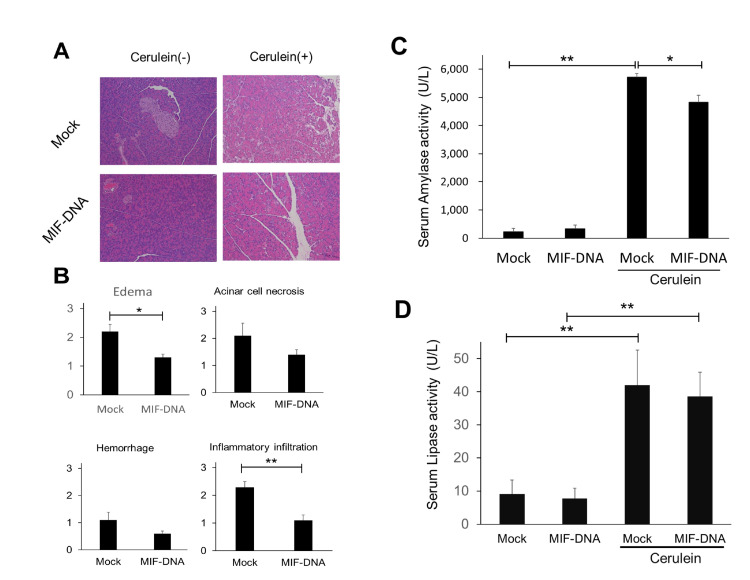
Effect of MIF-DNA vaccine on the severity of cerulein-induced pancreatitis. (A) Representative histological picture of mouse pancreas, original magnification ×100. (B) Histological scores. (C) Amylase activity in serum. (D) Lipase activity in serum. Each group: n = 5. Results were shown as means ± SE. *p < 0.05; **p < 0.01. MIF, migration inhibitory factor

We examined the effects of MIF-DNA vaccination on cerulein-induced pancreatitis. On histological examination, no morphological abnormality was observed in mock- or MIF-DNA vaccinated mice without administration of cerulein. When mice were treated with cerulein, mock-vaccinated mice displayed acinar cell injury, inflammatory infiltration, and intercellular edema in pancreas tissue (Figure 2A). In contrast, MIF-DNA vaccination improved these histological findings of pancreatitis in mice given cerulein (Figure 2A). In particular, the histological scores in intercellular edema and inflammatory infiltration were significantly (p<0.05 vs. mock- and cerulein-treated mice) reduced by MIF-DNA vaccination in mice with cerulein-induced pancreatitis (Figure 2B). On the other hand, seven times administration of cerulein markedly increased the levels of serum amylase activity (Figure 2C). MIF-DNA vaccination suppressed the increase of serum amylase activity induced by cerulein (p < 0.05 vs. mock- and cerulein-treated mice) (Figure 2C). However, the level of serum amylase activity in mock-or MIF-DNA vaccinated mice given cerulein was markedly high compared with the mice without administration of cerulein. On the other hand, there was no statistically significant difference in the serum lipase activity between mock-treated and MIF-DNA vaccinated mice given cerulein (Figure 2D).

Changes in the serum level of MIF by MIF-DNA vaccination in mice with cerulein-induced pancreatitis

**Figure 3:**
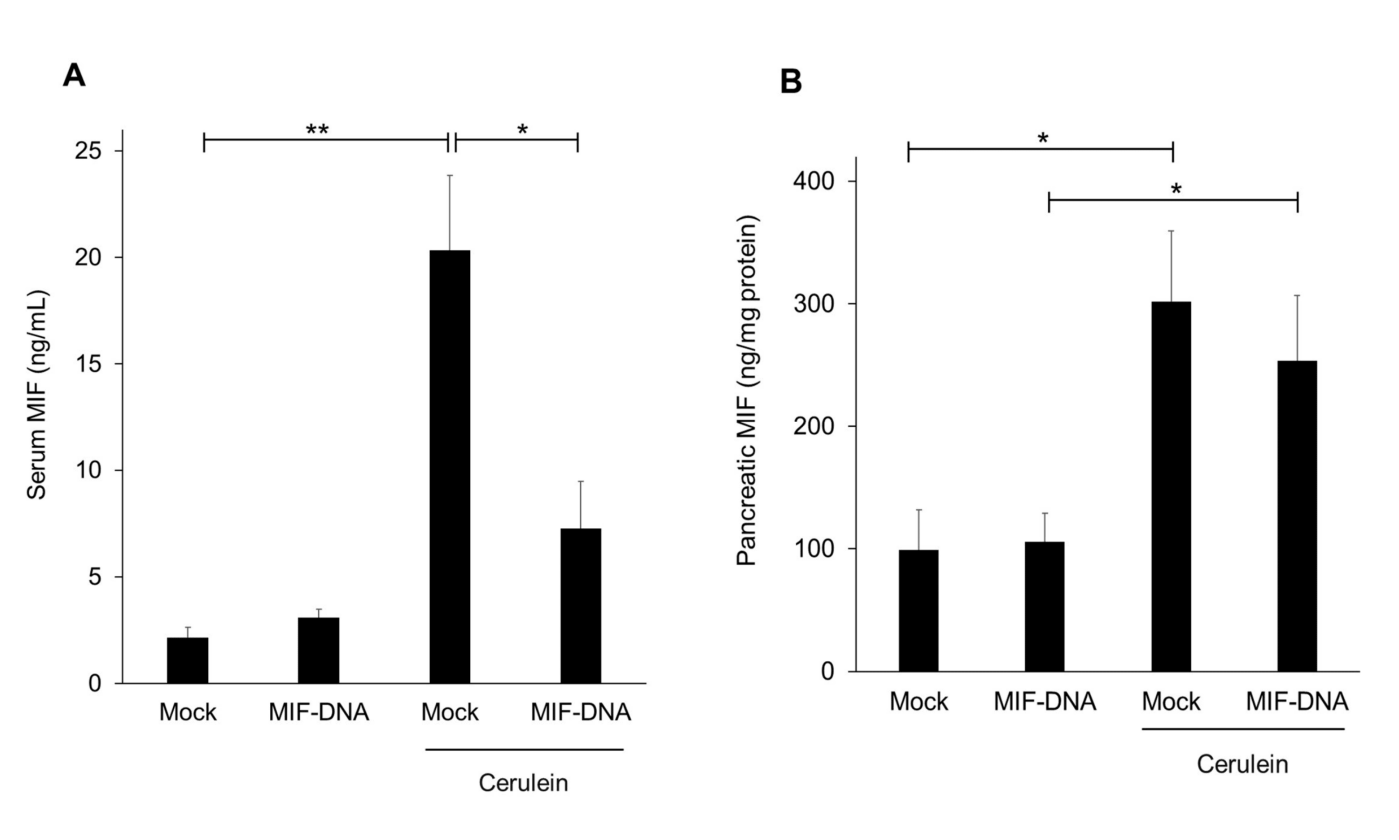
Effect of MIF-DNA vaccination on the levels of serum and pancreatic MIF in mice with cerulein-induced pancreatitis. (A) Serum level of MIF. (B) Pancreatic level of MIF. Results were shown as means ± SE. Each group: n = 5. *p < 0.05; **p < 0.01. MIF, migration inhibitory factor

The levels of MIF were markedly increased in the serum and pancreas tissue in cerulein-induced pancreatitis (Figure 3A). MIF-DNA vaccination inhibited the increased serum level of MIF in cerulein-induced pancreatitis (Figure 3A) (p < 0.05 vs. mock- and cerulein-treated mice). On the other hand, the upregulation of MIF protein level was not inhibited in the pancreas tissue of mice given cerulein (Figure 3B).

Changes in serum and pancreatic IL-1β by MIF-DNA vaccination in mice with cerulein-induced pancreatitis

**Figure 4:**
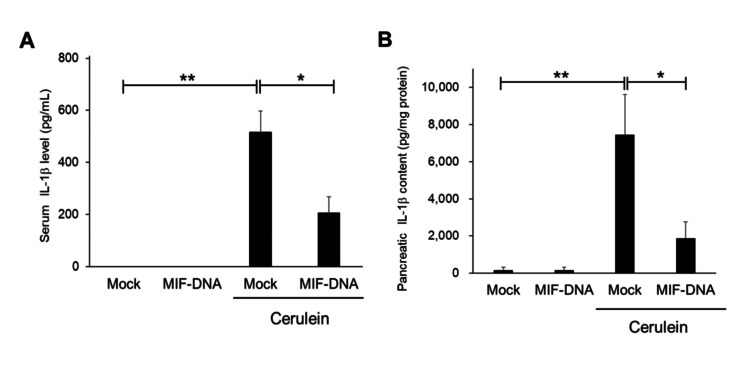
Effect of MIF-DNA vaccination on the serum and pancreatic levels of IL-1β in mice with cerulein-induced pancreatitis. (A) Serum level of IL-1β. (B) Pancreatic level of IL-1β. Each group: n = 5. Results were shown as means ± SE. *p < 0.05; **p < 0.01. MIF, migration inhibitory factor

The serum and pancreatic levels of IL-1β were increased in the serum of mice with cerulein-induced pancreatitis, while these increases were significantly suppressed (p < 0.05) in the MIF-DNA vaccinated mice compared with those in the mock-treated mice (Figure 4).

Changes in serum MCP-1 by MIF-DNA vaccination in mice with cerulein-induced pancreatitis

**Figure 5:**
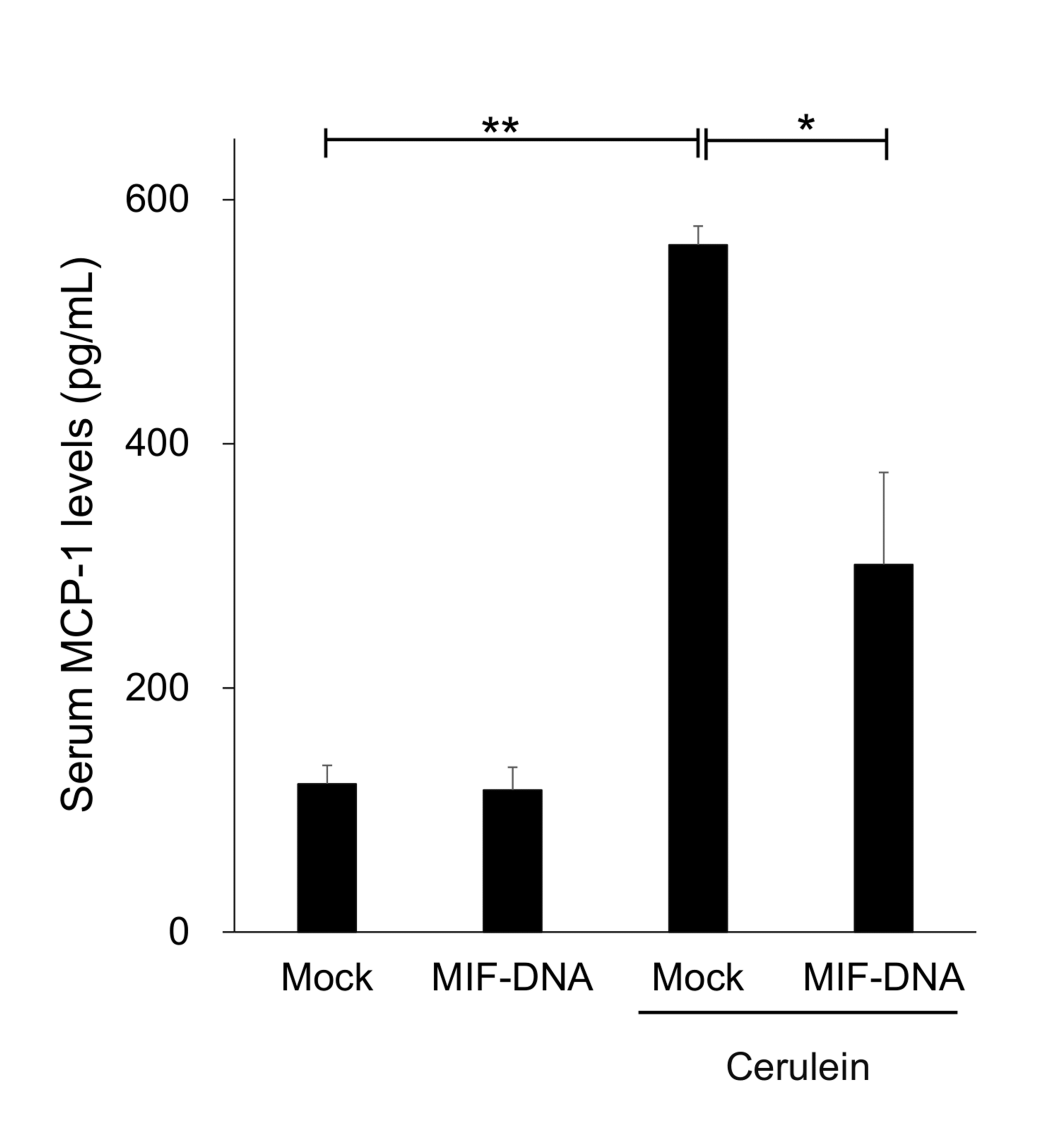
Effect of MIF-DNA vaccination on the serum level of MCP-1 in mice with cerulein-induced pancreatitis. Each group: n = 5. *p < 0.05; **p < 0.01. MCP-1, monocyte chemoattractant protein-1; MIF, migration inhibitory factor

The serum levels of MCP-1 were increased in mock-vaccinated mice given cerulein (Figure 5). MIF-DNA vaccination inhibited the increase of MCP-1 in the serum of mice treated with cerulein (p < 0.05) (Figure 5).

Changes in the pancreatic HSP70 level by MIF-DNA vaccination in mice with cerulein-induced pancreatitis

**Figure 6:**
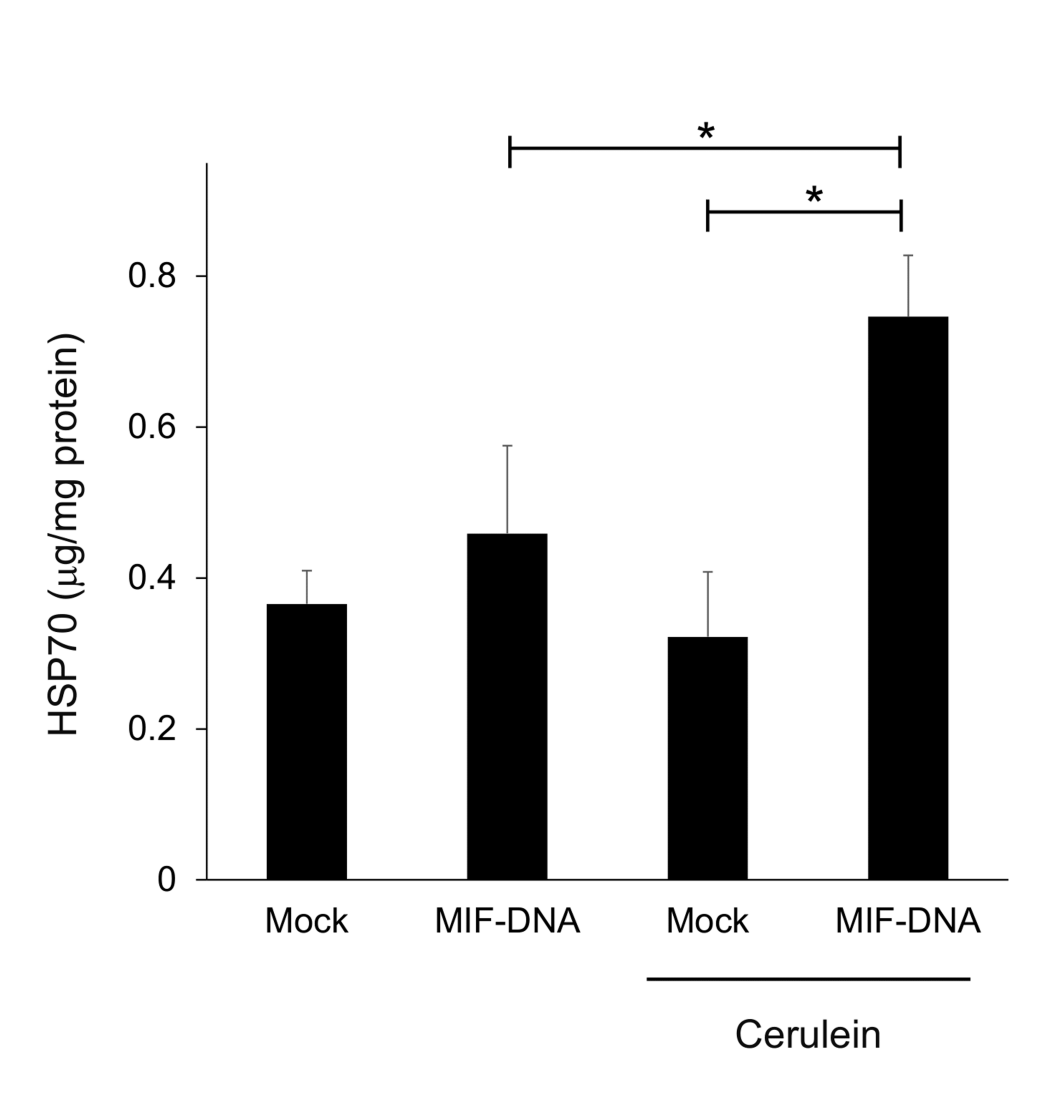
Effect of MIF-DNA vaccination on the pancreatic level of HSP70 in mice with cerulein-induced pancreatitis. Each group: n = 5. *p < 0.05. MIF, migration inhibitory factor

Before treatment with cerulein, there was no difference in the pancreatic HSP70 level between mock- and MIF-DNA vaccinated mice (Figure 6). Repeated administration of cerulein essentially unchanged the pancreatic HSP70 levels in mock-vaccinated mice (Figure 6). On the other hand, MIF-DNA vaccinated mice given cerulein showed the upregulation of HSP70 in the pancreas (p < 0.05 vs. mock-vaccinated mice with cerulein-induced pancreatitis) (Figure 6).

## Discussion

Pancreatitis is often progressive and leads to lethal outcomes; thus, a novel effective treatment is expected. MIF was upregulated in serum and pancreas in patients with pancreatic diseases, such as pancreatitis [10-12], and deletion or inactivation of MIF resulted in amelioration of the experimental pancreatitis [10,13,20]. MIF is recognized as a key regulator in inflammation and immune response. Inactivation of MIF by anti-MIF antibody or MIF inhibitor is effective for the treatment of various experimental inflammatory diseases [21]. Previous studies using MIF-DNA vaccine have shown the amelioration of inflammatory experimental models [15-17]. In this study, we demonstrated that MIF-DNA vaccination reduced the severity of cerulein-induced pancreatitis in mice. Furthermore, MIF-DNA vaccination inhibited the increase in serum MIF levels, suggesting that an anti-MIF antibody induced by the MIF-DNA vaccine may capture excessive extracellular MIF and suppress the development of pancreatitis. However, MIF-DNA vaccination did not reduce the pancreatic level of MIF in mice with cerulein-induced pancreatitis, suggesting that an anti-MIF antibody induced by MIF-DNA vaccination did not affect the expression of intracellular MIF in the pancreas. The meaning of the dissociation between extracellular and intracellular MIF levels is unclear; thus, further study is needed.

The secretion of pancreatic digestive enzymes is clinically used to evaluate the severity of pancreatitis. Cerulein is a cholecystokinin (CCK) receptor agonist, which activates the CCK receptor downstream signals, leads to product an excessive dose of digestive enzyme such as amylase and lipase [18]. Therefore, the increase in pancreatic digestive enzymes in serum helps us to evaluate the severity of cerulein-induced pancreatitis. On the other hand, some studies indicated that the inhibitory effect on cerulein-induced pancreatitis was independent of pancreatic digestive enzymes [22,23]. It is certain that the serum levels of pancreatic digestive enzymes are clinically useful for the evaluation of the severity of pancreatitis. In this study, MIF-DNA vaccination partially suppressed the upregulation of amylase activity in cerulein-induced pancreatitis in mice. The mechanism by which MIF-DNA vaccination suppresses the development of cerulein-induced pancreatitis may not be the inhibition of pancreatic digestive enzyme activation in mice.

Proinflammatory cytokines such as IL-1β are upregulated in pancreatitis [4]. Moreover, inhibition of IL-1β bioactivity improved the severity of experimental pancreatitis [24]. In this study, MIF-DNA vaccination suppressed the increase of IL-1β in the serum and pancreas of mice with cerulein-induced pancreatitis. Therefore, our results suggest that one of the mechanisms by which MIF-DNA vaccination inhibited the severity of pancreatitis is the down-regulation of IL-1β in mice treated with MIF-DNA vaccine.

Chemokines such as MCP-1 are regulated by MIF [25]. Especially, an anti-MCP-1 therapy protects against experimental pancreatitis [26]. In this study, increased serum level of MCP-1 in mice with cerulein-induced pancreatitis was inhibited by MIF-DNA vaccination, suggesting that MIF-DNA vaccination inhibits the development of cerulein-induced pancreatitis, at least partly via the suppression of MCP-1 upregulation.

HSP70 is one of the heat shock proteins and has cytoprotective effects against various stresses. Interestingly, our previous studies revealed that down-regulation of MIF enhanced the HSP70 upregulation in colon and stomach and inhibited the development of colitis and gastritis [27,28]. Additionally, HSP70 has a cytoprotective effect in experimental pancreatitis [29,30]. In this study, MIF-DNA vaccination upregulated the pancreatic HSP70 level in mice given cerulein, suggesting that the upregulation of pancreatic HSP70 level by MIF-DNA vaccination is protective against pancreatitis.

Our study has some limitations. First, our current experiments were a pre-treatment study. Thus, a study concerning the effect of the MIF-DNA vaccine after induction of pancreatitis is needed. Second, continuous inactivation of MIF by MIF-DNA vaccination may be immunosuppressive and harmful. In this study, MIF-DNA vaccination did not inhibit the serum MIF level under normal conditions. A study showing that MIF-DNA vaccination increases susceptibility to several infections is also needed. Furthermore, we could not investigate whether the upregulation of MIF antibody titer is sustainable. Long-term observation of the titer of serum MIF antibody induced by MIF-DNA vaccination is needed. In addition, we used only cerulein-induced pancreatitis. To develop clinical implications of MIF-DNA vaccination, we must investigate the effect of MIF-DNA vaccination using other models of pancreatitis. Although MIF-DNA vaccination may be useful for pancreatitis, further study is needed to resolve the limitations.

## Conclusions

In this study, treatment with the MIF-DNA vaccine protected mice from cerulein-induced pancreatitis. The histological findings of pancreatitis were inhibited in the MIF-DNA-vaccinated mice with cerulein-induced pancreatitis. Moreover, MIF-DNA vaccination inhibited the upregulation of serum MIF, serum and pancreatic IL-1β, and serum MCP-1, and increased the expression of pancreatic HSP70 level in cerulein-induced pancreatitis in mice. Although further study is needed, our results suggest that MIF-DNA vaccination has therapeutic potential for the treatment of pancreatitis. Furthermore, our results provide additional evidence that inhibition of MIF has the potential to be a therapeutic approach for pancreatitis.
